# A Biophysical Insight of Camptothecin Biodistribution: Towards a Molecular Understanding of Its Pharmacokinetic Issues

**DOI:** 10.3390/pharmaceutics13060869

**Published:** 2021-06-12

**Authors:** Andreia Almeida, Eduarda Fernandes, Bruno Sarmento, Marlene Lúcio

**Affiliations:** 1 INEB—Instituto Nacional de Engenharia Biomédica, Universidade do Porto, Rua Alfredo Allen 208, 4200-135 Porto, Portugal; andreia.almeida@ineb.up.pt (A.A.); bruno.sarmento@i3s.up.pt (B.S.); 2 i3S—Instituto de Investigação e Inovação em Saúde, Universidade do Porto, Rua Alfredo Allen 208, 4200-135 Porto, Portugal; 3 Instituto de Ciências Biomédicas Abel Salazar, Universidade do Porto, Rua Jorge Viterbo Ferreira 228, 4050-313 Porto, Portugal; 4 CF-UM-UP, Centro de Física das Universidades do Minho e Porto, Departamento de Física da Universidade do Minho, Campus de Gualtar, 4710-057 Braga, Portugal; eduardabfer@gmail.com; 5 CESPU, Instituto de Investigação e Formação Avançada em Ciências e Tecnologias da Saúde, Rua Central da Gandra 137, 4585-116 Gandra, Portugal; 6 CBMA, Centro de Biologia Molecular e Ambiental, Departamento de Biologia, Universidade do Minho, Campus de Gualtar, 4710-057 Braga, Portugal

**Keywords:** camptothecin, drug distribution, drug-membrane interaction, biophysical profiling, biomimetic models, partition coefficient, ADMET/PK prediction, small and wide-angle X-ray diffraction, fluorescence spectroscopy, human serum albumin (HSA)

## Abstract

Camptothecin (CPT) is a potent anticancer drug, and its putative oral administration is envisioned although difficult due to physiological barriers that must be overcome. A comprehensive biophysical analysis of CPT interaction with biointerface models can be used to predict some pharmacokinetic issues after oral administration of this or other drugs. To that end, different models were used to mimic the phospholipid composition of normal, cancer, and blood–brain barrier endothelial cell membranes. The logD values obtained indicate that the drug is well distributed across membranes. CPT-membrane interaction studies also confirm the drug’s location at the membrane cooperative and interfacial regions. The drug can also permeate membranes at more ordered phases by altering phospholipid packing. The similar logD values obtained in membrane models mimicking cancer or normal cells imply that CPT has limited selectivity to its target. Furthermore, CPT binds strongly to serum albumin, leaving only 8.05% of free drug available to be distributed to the tissues. The strong interaction with plasma proteins, allied to the large distribution (VD_SS_ = 5.75 ± 0.932 L·Kg^−1^) and tendency to bioaccumulate in off-target tissues, were predicted to be pharmacokinetic issues of CPT, implying the need to develop drug delivery systems to improve its biodistribution.

## 1. Introduction

Camptothecin (CPT) is an alkaloid isolated from the Chinese tree *Camptotheca acuminata* and it is a promising agent against cancer [1]. CPT is a pH dependent molecule that can exist in two different forms: the lactone form, which is stable at pH <5.5 and biologically active or the carboxylate form, which is stable at pH >9 and inactive. CPT inhibits the topoisomerase I, which is a nuclear enzyme implied in the DNA replication, by binding the topoisomerase I-DNA complex, avoiding the replication process and, consequently, leading to the cell death [2,3]. Its mechanism of action only occurs in the S-phase of the cell cycle. Interestingly, cancer cells spend more time in the S-phase compared to healthy cells, which means that CPT is more likely to bind to cancer cells and therefore, exert its mechanism of action. This selectivity is one of the characteristics that makes CPT a potential drug for cancer treatment.

However, CPT has some disadvantages that make its effectiveness less than expected, as the case of the pH-dependency and the poor water solubility.

CPT at physiologic pH is hydrolyzed and the molecule is partially converted into the carboxylate form, where the ring is opened and, thus biologically inactive. The lactone active form is present in the biological fluids at a very low concentration. It is described that after 2 h in contact with human blood, only 5% of the CPT lactone form remains [3], which makes its effectiveness against cancer impossible. In addition, in the human plasma, the carboxylate form is the most abundant microspecies, which has great affinity to the human serum albumin (HSA). Once CPT and HSA are bound, it is no longer possible to convert the molecule into its lactone form [4]. However, the stability of CPT can be improved once the molecule bounds to membranes, which avoids its hydrolysis [5]. Thus, it is very important to understand the interaction of CPT when in contact with cellular membranes, proteins, or biological fluids to predict its behavior for further in vitro and in vivo studies.

The pharmacokinetic (PK) parameters of a drug are dependent on its freely or bound form present in the biological fluids, as well as, the therapeutic efficacy is tightly related to the affinity to plasma proteins [6]. However, drug design goes beyond drug–protein interactions. The membrane location and orientation, and the membrane affinity and distribution dictated by the partition coefficient (log P) and distribution coefficient (log D) of a drug are parameters used to predict the transport, distribution, accumulation, and therapeutic effects of compounds in vivo [7]. Thus, the determination of the drug ADMET profile (absorption, distribution, metabolism, elimination, and toxicity) through the study of molecular interactions plays a key role on the drug PK [8,9]. Further, 1,2-dioleoyl-sn-glycero-3-phosphatidyl-choline (DOPC), 1,2-dioleoyl-sn-glycero-3-phospho-l-serine (DOPS), 1,2-di-oleoyl-sn-glycero-3-phosphoethanolamine (DOPE), sphingomyelin (SM), or cholesterol (CHOL) are widely used to mimic the performance of biological membranes in vitro since they represent some of the main lipid components of the biological membranes [10]. Additionally, while using computational in silico models, it is possible to determine these PK key factors and evaluate if a molecule is worth to progress for in vitro and/or in vivo studies [11,12].

The interaction of CPT with membrane models and with plasma proteins has already been addressed in previously reported studies [13,14,15]. However, the reports in literature are sparse, use few techniques, and do not provide a comprehensive overview of the molecular interactions established between the drug and biological interfaces throughout its physiological path. In the literature, it is possible to find a few studies of binding of CPT to membranes of DMPC, DMPG, DOPC, or DOPG and CPT binding to HSA or bovine serum albumin (BSA) [16,17,18]. The purpose of this research is to give a more complete overview of the interactions between CPT and biointerfaces after a putative oral administration of this drug and simultaneously provide researchers with an example of in vitro profiling of a known drug, used as a model, that can be applied to predict/explain some aspects of the biological behavior of other drugs in vivo. Therefore, the current study is intended to go further than the reported CPT/membrane interaction studies by evaluating the distribution of CPT in more complex membrane models mimetic of cancer or normal cells and endothelial membranes of the blood–brain barrier (BBB). These different membrane models will permit using more accurate in vitro membrane distribution values to be used together with theoretical models and predict drug distribution at therapeutic targets as well as at off-target sites. Moreover, this study also focuses on the biophysical aspects of the interaction of CPT with membrane models, which to our knowledge, have not been addressed, such as the location/orientation of CPT within the membrane, CPT effect on the membrane fluidity and the effect of the drug on the membrane structure, and phospholipid packing at different lipid phases. To achieve the aims proposed, a detailed study of the CPT physicochemical properties was conducted and several biophysical techniques were employed, such as dynamic light scattering (DLS), derivative UV-Vis spectroscopy, fluorescence spectroscopy, intrinsic and extrinsic fluorescence quenching, or synchrotron small and wide-angle X-ray scattering (SAXS and WAXS).

Since CPT is used as a model drug, the results obtained can help to understand what the drug’s possible responses will be when, after administration, it is exposed to different biointerfaces. This approach allowed to accurately predict some challenges of CPT biodistribution and to purpose drug delivery strategies to overcome these challenges. Therefore, the results here presented can be used by researchers as an example of an in vitro profile to predict the behavior of drugs in vivo, at early stages of formulation development, avoiding the unnecessary use of cells and/or animals in agreement with the EU principles (Directive 2010/63/EU).

## 2. Materials and Methods

### 2.1. Materials

Camptothecin (CPT) was acquired from Jinlan Pharm-Drugs Technology Co., Ltd. through Hangzhou ROYAL Import & Export Co., Ltd. (Hangzhou, China). The lipids 1,2-dioleoyl-*sn*-glycero-3-phosphocholine (DOPC), 1,2-dioleoyl-sn-glycero-3-phospho-l-serine (DOPS), 1,2-di-oleoyl-sn-glycero-3-phosphoethanolamine (DOPE), cardiolipin (CL), the lipid labeled probe 1,2-dipalmitoyl-sn-glycero-3-phosphoethanolamine-*N*-(7-nitro-2-1,3-benzoxadiazol-4-yl) (NBD-PE), and the brain polar lipids (BPL) were obtained from Avanti Polar Lipids, Inc. (Instruchemie, Delfzijl, The Netherlands). Egg phosphatidylcholine (EPC), sphingomyelin (SM), and cholesterol (CHOL) were purchased to Sigma-Aldrich Química, S.L. (Sintra, Portugal). All reagents were the highest purity available and were used without further purification. Membrane models of cancer cells were prepared in acetate buffer (pH 5.8) and membrane models of normal cells were prepared in HEPES buffer (pH 7.4). All other solutions were prepared with water from a Milli-Q plus system with specific conductivity less than 0.1 µS cm^−1^.

### 2.2. In Silico Predictors of Drug-Profiling

Relevant drug physical-chemical properties (e.g., number of hydrogen bonds, polar (PSA) and van der Waals (VWSA) surface area, solubility, LogD and LogP, and ionization) were calculated using the software MarvinSketch^®^ (ChemAxon, Budapest, Hungary). From these physicochemical properties and based on reported molecular descriptors, drug-profiling was established to give insights about formulation design challenges for CPT delivery [19].

### 2.3. Membrane Models Preparation

Single-lipid liposomal membrane models composed of DMPC, or lipid mixture liposomal membrane models mimetic of breast cancer cell membrane—DOPC (25%), CHOL (15%), EPC (31.8%), DOPS (17%), DOPE (8%), cardiolipin (2.5%) and SM (0.7%)—mimetic of cell membrane—DOPC (45%), DOPE (20%), DOPS (20%), CHOL (10%), and SM (10%)—and, blood–brain barrier (BBB) membrane constituted by the BPL—phosphocholine (PC) (12.6%), phosphatidyl ethanolamine (PE) (33.1%), PI (4.1%), phosphatidyl serine (PS) (18.5%), and PA (0.8%) were prepared by the classical thin film hydration method [20]. Briefly, lipids were co-dissolved in chloroform:methanol (8:2, v/v) and the solvents were evaporated under a stream of nitrogen in a rotary evaporator (Buchi R-200; Sigma-Aldrich Corp., Buchs, Switzerland). The resultant dried lipid film was hydrated with buffer at temperature of 40 ± 0.1 °C and lipid colloidal dispersions were formed by alternating vortex and thermostatic bath (40 ± 0.1 °C). Subsequently, unilamellar lipid model systems were obtained from extruding (Lipex^®^ extruder, Tranferra Nanosciences, Burnaby, Canada) colloidal dispersions through polycarbonate filters with a pore diameter of 100 nm (Millipore SAS, Molsheim, France).

For the location studies, NBD-PE labeled lipid model systems were used, prepared by the lipid film hydration method above described, followed by extrusion. In the lipid film preparation process, the probe was co-dried with the lipid in a ratio of 300:1 (lipid:probe) to prevent changes in the structure of the membrane [21].

### 2.4. Drug Distribution Coefficient in Membrane/Aqueous Systems Studied by Derivative Spectroscopy

The distribution coefficient (LogD) of CPT between membrane and aqueous buffered solution (pH 5.8 for breast cancer cell membrane model and pH 7.4 for cell membrane model and BBB membrane model) was determined by fluorescence spectroscopy and derivative UV-Vis spectroscopy. For each model, two groups of suspensions were prepared: the samples and references. The samples contained a fixed concentration of CPT (2 × 10^−5^M) and increasing concentrations of lipid suspension (0 to 1 × 10^−3^ M for absorbance or 3 × 10^−3^ M for fluorescence). The references were identically prepared in absence of CPT. Then, all the suspensions were incubated during 30 min at 37.0 ± 0.1 °C. After incubation, the absorption spectra of samples and references were plotted in the 230–500 nm range, on a Perkin-Elmer Lambda 45 UV-Vis, accordingly to a well-established protocol [22] at 37.0 ± 0.1 °C. The fluorescence spectra were also measured on a Perkin-Elmer LS-50B spectrofluorimeter. The emission spectra were obtained in 380–480 nm range with λ_excitation_ = 250 nm and the excitation spectra were recorded in 220–400 nm range with λ_emission_ = 430 nm. The excitation and emission slits were adjusted to 6 nm. The second and third derivative spectra of the absorbance UV-Vis spectra were determined to improve the resolution of spectral bands and to eliminate the spectral interferences due to light scattered by the lipid suspensions. Representing the λ_max_ or λ_min_ values of the 1st, 2nd, or 3rd derivative spectra as a function of the concentration of lipid model systems ([Membrane]), a non-linear regression was obtained from which it was possible to determine the distribution coefficient (D):

(1) $$\frac{d^{n}S_{T}}{d\lambda^{n}}=\frac{d^{n}S_{a}}{d\lambda^{n}}+\frac{\left(\frac{d^{n}S_{m}}{d\lambda^{n}}-\frac{d^{n}S_{a}}{d\lambda^{n}}\right)\cdot D\cdot \left[Membrane\right]\cdot V_{m}}{1+D\cdot \left[Membrane\right]\cdot V_{m}}$$

where S_T_, S_a_, and S_m_ stands respectively for total (T) absorbance of the drug in membrane (m) and aqueous media (a). The membrane model systems’ concentration, [Membrane] expressed in mol∙L^−1^, is multiplied by the lipid molar volume in L∙mol^−1^ (V_m_) of each membrane model to obtain a dimensionless value of D, from which logD is calculated.

Because CPT is a fluorescent compound with spectral variations due to its distribution between the lipid/aqueous phases (e.g., λ shifts), logD of CPT can also be calculated using fluorescence spectroscopy [23]. The fluorescence emission intensity, *I,* can be used to calculate the distribution D, and the correspondent logD, through Equation (2):

(2) $$I_{T}=I_{a}+\frac{\left(I_{m}-I_{a}\right)\cdot D\cdot \left[Membrane\right]\cdot V_{m}}{1+D\cdot \left[Membrane\right]\cdot V_{m}}$$

where the fluorescence emission intensity (I_T_) of CPT distributed in lipid accounts for the fluorescence emission intensity contributions of the drug distributed in the membrane (I_m_) and aqueous (I_a_) phases which can be related to D and then logD values [23].

### 2.5. Drug Location in Membrane/Aqueous Systems Studied by Steady-State Fluorescence Quenching

The cancer cell membrane model was labeled with the NBD-PE probe. The fluorescence of the fluorophore was monitored by steady-state fluorescence studies to predict the CPT location at the membrane level. Increasing concentrations of CPT (0 to 4.0 × 10^−5^ M) and a fixed concentration of the labeled cancer membrane model system (3.0 × 10^−2^ M) were used. Steady-state fluorescence emission (Perkin-Elmer LS-50B spectrofluorimeter, Waltham, USA) was obtained using λ_excitation_ = 360 nm (maximum excitation of CPT). Fluorescence excitation (300–400 nm) and emission spectra (500–600 nm) were recorded using slits of 9 and an integration time of 10 s was used. The extent of fluorescence quenching induced by CPT in NBD-PE probe was evaluated by Stern–Volmer constant (K_SV_) obtained by fitting data to the Stern–Volmer linear plots:

(3) $$\frac{I_{0}}{I}=1+K_{SV}\cdot {\left[CPT\right]}_{m}$$

where I and I_0_ are the steady-state fluorescence, respectively, in the presence or in the absence of CPT_._ [CPT]_m_ is the membrane concentration of CPT calculated as described elsewhere [24]:

(4) $${\left[CPT\right]}_{m}=\frac{K_{d}\cdot {\left[CPT\right]}_{T}}{K_{d}\cdot \alpha_{m}+\left(1-\alpha_{m}\right)}$$

where K_d_ is the distribution coefficient of CPT in the membrane model system (calculated as described in Equation (1)), [CPT]_T_ is the total concentration of CPT, and α_m_ is the membrane volume fraction.

The efficacy of CPT to quench the fluorescence of the probe was evaluated by the bimolecular constant (K_q_) [25]:

(5) $$K_{q}=\frac{K_{SV}}{\tau_{0}}$$

where τ_0_ is the unquenched lifetime. All fluorescence intensity data were corrected from absorption and inner filter effect [25].

### 2.6. Plasma Protein Binding Evaluation Using Steady-State Fluorescence Quenching

The fluorescence excitation and emission spectra of increasing CPT concentrations (0 to 3 × 10^−4^ M) with a constant HSA concentration (2.0 × 10^−6^ M) were acquired at 37.0 ± 0.1 °C (Perkin-Elmer LS-50B spectrofluorimeter, Waltham, USA), with excitation at 280 nm and emission at 342 nm, each with 5 nm slits. The following binding isotherm equation can be used to explain the quenching of HSA fluorescence caused by increasing CPT concentrations [25]:

(6) $$\%\;Quenching=\frac{y_{máx}\cdot n}{1+\frac{K_{diss}}{\left[CPT\right]}}$$

where y_max_ is the maximum fluorescence quenching measured, n is the number of binding sites of HSA to CPT, and K_diss_ is the dissociation constant (i.e., the inverse of binding constant, K_bind_). In addition, the following equation expresses the relationship between the K_diss_ value and the Gibbs free energy (ΔG) of complex binding:

(7) $$\Delta G_{bind}=R\cdot T\cdot \ln(K_{bind})$$

where R is the ideal gas constant and T is the temperature in Kelvin (K).

### 2.7. Small- and Wide-Angle X-ray Scattering Studies at the Synchrotron

Membrane model systems (DMPC) in the presence or absence of CPT were put into 1.5 mm diameter X-ray clear glass capillaries for X-ray scattering studies (Hilgenberg, Malsfeld, Germany). Capillaries were sealed with a flame and kept at 4 °C. SAXS and WAXS experiments were carried out at the Austrian SAXS/WAXS beamline at the synchrotron light source ELETTRA (Trieste, Italy), using monochromatic synchrotron radiation with a wavelength of 1.54 nm and an X-ray energy of 8 keV. SAXS and WAXS patterns were captured at locations that covered the normal diffraction spacing spectrum (s = 2 sin θ/λ, where λ is the wavelength and 2θ is the scattering angle) of interest using a 2D Pilatus3 1M and a 2D Pilatus 100K detector device, respectively, with a pixel size of 172 μm. The lamellar peaks of silver behenate (SAXS) and *p*-bromo benzoic acid (WAXS) were used as criteria to calibrate the diffraction spacings. Static exposures were taken below and above the main transition temperature as controlled by a thermostated water bath (stability ±0.1 °C; Unistat CC, Huber, Offenburg, Germany) to obtain the diffraction patterns of normal lipid phases ($L_{\beta }$, ripple phase $P_{\beta },$ and $L_{\alpha }$) and the effect of CPT in those phases. The data were analyzed in the same way as they had been in previous studies [24].

### 2.8. Studies on Dynamic and Electrophoretic Light Scattering

Dynamic light scattering (DLS) was used to assess the influence of CPT addition in the main phase transition temperature and cooperativity (B) of the DMPC membrane model system [24,26]. As a result, the light scattered (mean count rate, MCR) by membrane system (5.0 × 10^−2^ M) in the absence and presence of CPT (2.0 × 10^−2^ M) was measured as a function of temperature (T) between 30 and 55 °C (with 1 °C intervals and 2 min of equilibration time). A sigmoidal function based on a modified Boltzmann equation was used to fit the results [24,26]:

(8) MCR=b1+m1·T+b2−b1+m2·T−m1·T1+10B·(1T−1Tm)

where *m_1_* and *m_2_* are the slopes obtained by fitting the data linearly before and after *Tm*, respectively, and *b_1_* and *b_2_* are the corresponding y-intercepts. All DLS and ELS experiments were carried out in a Zetasizer Nano ZS with disposable polystyrene cells and a dipcell (ZEN1002) for the ELS studies.

### 2.9. Modelling Biodistribution Using In Vitro Parameters

The *logD* value obtained can be used with in silico descriptors to predict many biodistribution parameters using mathematical methods. As a result, the value of logD was used to predict CPT bioaccumulation (as expressed by the bioaccumulation constant, K_bioaccumulation_), which is associated to tissue blood flow (Q) and its volume (V) [27]:

(9) $$K_{bioaccumulation}=\frac{Q}{V\cdot \log D}$$

To determine if the drug is stored in adipose tissue, the adipose store index (ASI) of CPT was calculated using the following equation [28]:

(10) ASI=1.81·logD−logD+0.40

Furthermore, the obtained distribution coefficient in the BPL model system—logD_(BBB)_—can be compared to logBB, according to Waterbeem and Kansy [29]. The logarithm of the ratio of drug concentrations in brain and blood is known as logBB, and it represents the drug’s relative affinity variations between plasma proteins and brain tissue [30]:

(11) $$\log BB=0.388\cdot \log D_{BBB}-0.00618\cdot V_{m}+1.359$$

where $V_{m}$ is the membrane model system’s molar volume, determined from the individual lipid volumes [25].

The steady-state volume distribution (VD_SS_) is one of the most useful PK criteria for describing a drug’s biodistribution [31]. The effect of the PPB, permeability, partitioning, and active transport on the drug’s physiological distribution is translated by this predictor. Based on human clinical PK evidence of a wide set of drugs (670 drugs), the first physiological statistical model of VD_SS_ for predicting the biodistribution of neutral and basic drugs was proposed [32]:

(12) VDSS=VP·(1+REL)+fu·VP(VEVP−REL)+VR·fufut

where V_P_ and V_E_ are the plasma and extracellular fluid volumes with corresponding value in human of 0.0436 and 0.0151 L∙Kg^−1^; R_E/I_ represents the ratio of extravascular and intravascular proteins, and is strictly referred to the distribution of albumin, assuming an approximate value of 1.4; V_R_ is defined as the physical volume into which the drug distributes minus the extracellular space (0.380 L∙Kg^−1^); f_u_ and f_ut_ are, respectively, the unbound drug fraction in plasma and the unbound fraction in tissues [32].

The f_u_ values can be obtained by the following equation [33]:

(13) $$f_{u}=1-\frac{PPB}{100}$$

where PPB can be obtained from the in vitro values of K_bind_ [33]:

(14) $$PPB=\frac{100}{1+\frac{1}{C_{P}\cdot K_{bind}}}$$

where C_P_ is the physiological plasma protein concentration (750 μM) [33]. 

Lastly, f_ut_ can be obtained by the following equation [32]:

(15) $$\log f_{ut}=-0.0289-0.1739\cdot \log D-0.8324\cdot f_{i(7.4)}+1.0400\cdot \log f_{u}$$

where f_i(7.4)_ is the fraction of ionized drug at pH 7.4 which has been calculated considering the pH hydrolysis and conversion of CPT from lactone neutral form to the anionic form carboxylate reported [34].

### 2.10. Statistical Analysis of Data

All data were expressed as mean ± standard deviation of three independent experiments. Multiple comparisons were performed using a two-way variance analysis (ANOVA) with a Sidak’s multiple comparisons test or one-way ANOVA with the Student–Newman–Keuls as a post-test. Values of * *p* < 0.05; ** *p* < 0.01; *** *p* < 0.001 have been considered statistically significant.

## 3. Results

### 3.1. In Silico Analysis of CPT Physicochemical Properties

**Figure 1:**
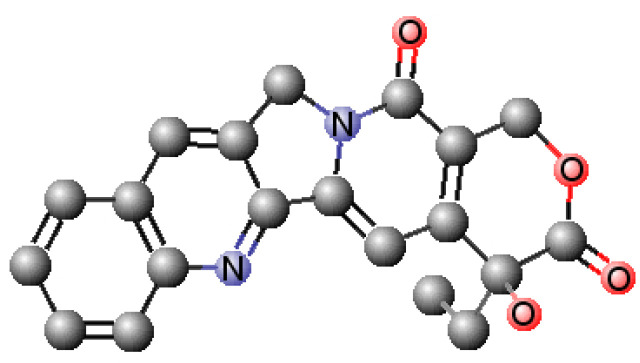
3D chemical structure of CPT generated with ‘Chemicalize’ tool from Chemaxon^®^ software.

In silico methods are widely used to perform systematic analysis of the physicochemical and biopharmaceutical properties of potential drugs. Understanding the challenges that a drug may present is critical and can lead to new methods in the formulation manufacturing in order to overcome these challenges. Some pertinent physicochemical properties of CPT (Figure 1) were predicted in silico (Table 1). Following that, conclusions regarding its ionization, lipophilicity, permeability, and solubility were drawn, as well as its classification according to the Biopharmaceutical Classification System (BCS).

Taking into consideration the Lipinski ‘rule of five’, a compound is more likely to have poor permeability if at least two of the following parameters are observed: LogP > 5, MW > 500 Da, H donors > 5, and H acceptors > 10 [35]. Since CPT does not follow any parameters of the Lipinski rule, it is thus expected to have good permeability. Moreover, CPT demonstrated to have low polarizability (PSA = 79.73 Å^2^), which is correlated with a good permeability profile at the cell membranes [36]. However, when its lipophilicity is observed, we can conclude that CPT is moderate lipophilic (0 < logP < 3) [37] and presents poor aqueous solubility (0.0559 mg∙mL^−1^). These factors, despite not being contemplated by the Lipinski rule, do not facilitate the permeability of the compound, which is in agreement with other published work [38]. Additionally, the permeability of a compound can be related with the concentration used. Indeed, CPT permeability is dependent of the concentration, being more permeable at lower concentrations (5 µM) and, even so, its Papp is relatively low (3 × 10^−5^ cm∙s^−1^) [38]. Moreover, from the in silico evaluation of the physicochemical properties of a molecule, it is not possible to predict if the molecule will suffer hydrolysis, as in the case of CPT. Since CPT is pH-dependent and not stable at neutral pH, it can easily suffer hydrolysis and, at physiologic pH, be converted in the carboxylate form, which is a more aqueous soluble, less permeable, and it is not biological active to bind the topoisomerase I. None of these aspects could be predicted only from the in silico evaluation of CPT. Indeed, if we would only rely on in silico calculations, CPT would be classified by the BCS as Class II. However, as previously described, this prediction does not take into consideration the stability of the molecule and the pH-dependency, thus, the BCS classification of CPT should be the one officially adopted, that is, a Class IV compound [39].

Regarding the CPT ionization character, the obtained in silico pKa values (Table 1) are similar to the pKa values found for CPT analogues (pKa = 2.32 and 9.15) [40,41].

From the analysis made for CPT, it is possible to conclude that using only in silico approaches is not always a straightforward or reliable method to predict biodistribution. Recent developments in biodistribution prediction and PK profiling recommend the use of in vitro approaches, complemented by in silico analysis and models, to predict tissue distribution using physicochemical properties [32,42,43,44]. Therefore, it is important to evaluate other parameters, like the interaction with biological membranes, in order to predict the molecule behavior in the biological fluids. These predictions can be further improved by considering in vitro data and binding of drugs to plasma proteins [32,42,43,44]. Therefore, in the following sections, in vitro studies of the interactions of CPT with models of biointerfaces will be conjugated with in silico properties and theoretical models to evaluate CPT biodistribution. This approach may be very helpful when formulating new oral delivery dosage forms of CPT, aiming to understand how to maintain drug’s stability and biological function, at the same time improving its aqueous solubility and mucus diffusion.

### 3.2. Distribution and Location of CPT in a Membrane/Aqueous System

The distribution coefficient (logD) between lipid and aqueous phases, which allows estimation of drug lipophilicity and distribution in hydrophobic and hydrophilic microenvironments, is the first physicochemical property that provides strong evidence to support drug distribution in body tissues [23].

**Figure 2:**
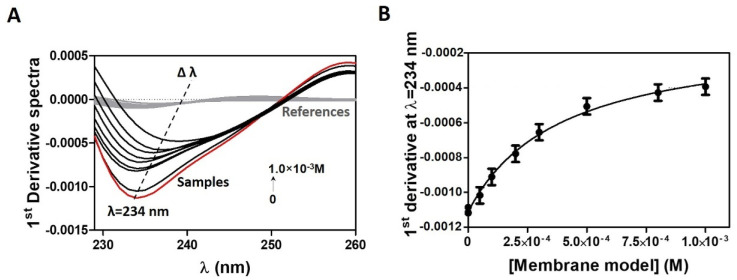
(**A**) First derivative of CPT (2 × 10^−5^) absorption spectra with increasing concentrations of cancer membrane models (0 to 1.0 × 10^−3^ M) in acetate buffer pH = 5.8. In red, are represented the absorption spectra of CPT in the absence of the cancer membrane models. With the addition of increasing concentrations of membrane model (samples represented in black), there is a shift in λ values. In grey, are represented the derivative spectra of the references containing only membrane models. (**B**) Nonlinear fitting of derivative absorbance values at λ = 234 nm as a function of membrane model concentration.

Accordingly, the logD of CPT was determined in cancer cell membrane models, cell membrane models, and BBB membrane models in order to simulate the various membrane barriers encountered by this drug during its biological distribution. The study in cancer cell membrane models was carried out at a pH of 5.8 to mimic the acidic microenvironment of tumors (pH 5.6 to 6.8), which is a hallmark of malignant cancer cells and is caused by glycolysis in cancer cells, hypoxia, and insufficient blood perfusion [45]. The studies in cell membrane models were carried out at pH 7.4, representative of blood and healthy tissues. Figure 2 shows an example of absorbance spectra and the subsequent derivative method for data analysis.

The first derivative spectra were calculated from the CPT absorbance spectra experimentally obtained for increasing concentrations of the membrane model (Figure 2A), as this eliminates the effect of Rayleigh dispersion, which is higher for shorter wavelengths [23]. The use of first derivative spectra eliminates light scattering interference and improves spectral resolution, revealing more details about the CPT–lipid interaction. Indeed, at the derivative minimum, a shift of λ (of about 25 nm) is visible, indicating CPT distribution into lower polarity environments (distribution of CPT into the lipid phase) [23], with a decrease in the intensity of the bands as the lipid concentration increases. There is also an isosbestic point observed, indicating the presence of a balance between two CPT forms (interacting with the lipid medium and free in aqueous buffered medium) and the elimination of light-scattering interference [23]. Data were plotted against the respective membrane model concentration (Figure 2B) using maximum or minimum values from derivative spectra (e.g., 234 nm), and the resulting data points were fitted to Equation (1) (Figure 2B).

**Figure 3:**
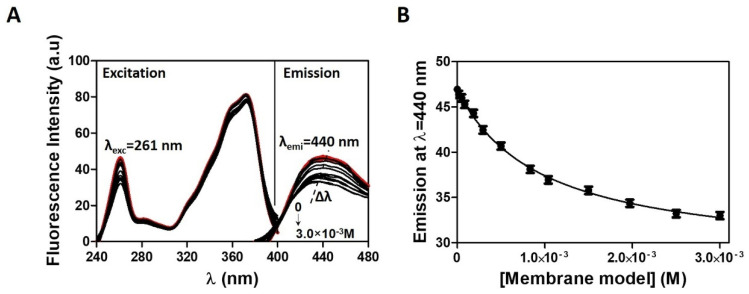
(**A**) Fluorescence emission spectra of CPT (2 × 10^−5^) with increasing concentrations of cancer membrane models (0 to 3.0 × 10^−3^ M) in acetate buffer pH = 5.8. In red, are represented the absorption spectra of CPT in the absence of the cancer membrane models. With the addition of increasing concentrations of membrane model (samples represented in black), there is a shift in λ values. (**B**) Correspondent nonlinear fitting (Equation (1)) of fluorescence emission values at λ_max_ = 440 nm as a function of membrane model concentration.

The determination of logD in membrane/aqueous phase by fluorescence methods is more advantageous over UV-Vis spectrophotometric methods, since the light scattering caused by the lipid media is negligible and was subtracted from each sample spectrum to cancel out any contribution (Figure 3A). With exception of the BBB membrane model, where the higher membrane scattering invalidated the use of fluorescence method, it was possible to plot the maximum emission values against the respective membrane model concentration (Figure 3B) without having to derive the spectra, and the resulting data points were fitted to Equation (2).

D values were calculated based on both absorbance or fluorescence nonlinear fittings and used to express membrane distribution of CPT as logD values (Table 2).

When the logD values for the membrane models and pH values mimetic of normal and cancer cells are compared, regardless of the method used (derivative UV-Vis spectroscopy or fluorescence spectroscopy), it can be concluded that CPT can distribute between the membrane and aqueous phases with no significant differences, with an average logD value of 2.89 ± 0.23, which is typical of intermediate to lipophilic molecules (0 < logD < 3) [28]. A drug’s ability to distribute between membrane/aqueous media indicates that it can penetrate cell membranes through the phospholipid polar head region, diffuse through lipophilic hydrocarbon chains, and emerge into the inner region of phospholipid polar headgroups, which determines its body distribution [46,47]. Therefore, these logD results also indicate that CPT is well distributed within lipid membranes and can thus have large body distribution.

The drug’s affinity for a tissue or organ is also determined by the drug’s distribution and accumulation in the tissue. As shown in Equation (9), the factors that determine the distribution coefficient of a drug into an organ are related to the blood flow to the organ, the organ’s volume, and the drug’s distribution into the tissues. Based on the experimental results of CPT distribution in normal membrane cells (Table 2) and the described values of blood flow and volume of different organs, it is possible to predict CPT off-target distribution using reported drug profiling models [27] by the following tissues: adrenal glands (61.44 %), thyroid (30.72%), kidneys (3.07%), and heart (3.07%).

Aside from estimating drug distribution in the off-target organs mentioned above, the CPT adipose storage index (Equation (10)) was calculated to be 2.53 ± 0.12, which accounts for drug distribution in adipose tissue, indicating a high distribution in these tissues comparable to other neutral drugs such as clobazam [28]. Furthermore, the brain accumulation index (Equation (11)), which accounts for the distribution of the CPT in the brain tissues, was calculated, using the logD values in BBB endothelial membrane, to be LogBB = 2.76 ± 0.06, which suggests potential BBB permeability [48].

The lipophilicity of drugs is typically expressed as a partition of the drug in the octanol/water system, and the reported logP value (value of drug distribution at a pH value where the drug is in its neutral form) of CPT in the octanol/water system was 1.73 ± 0.08 [18,49,50] (Table 2), whereas the in silico calculated logD (at pH 7.4 or 5.8) and logP values in the octanol/water system using Chemaxon^®^ software were 1.52 (Table 1), i.e., significantly lower than the distribution logD values determined in vitro in the membrane/aqueous system (Table 2).

The fact that octanol does not mimic the amphiphilic nature of membranes is a significant disadvantage of theoretical models based on octanol/water biphasic systems [9,23,51]. Contrastingly, because they mimic the hydrophobic core and polar surface of biomembranes, lipid/water systems have been developed as improved cell membrane models and are used to represent drug distribution in biological media [52]. Hydrophobic, H-bonding, dipole-dipole, and electrostatic interactions between drug and membrane are considered using biomimetic membrane models, whereas the octanol/water system can only model nonpolar interactions [53,54]. Since interactions between the drug and the polar headgroups of membrane lipids are not considered when octanol is used, it is understandable that CPT partitioning in octanol/water systems yielded lower values than partitioning in membrane/water systems. The use of membrane/water systems rather than octanol/water systems is especially important for amphoteric compounds or highly ionized drugs that are charged at physiological pH, and it has been observed that, aside from non-ionic drugs, partitioning values in octanol/water systems do not correlate well with experimental values [22,55].

When the results for the different mimetic systems of normal membrane cells are compared, the CPT distribution between the membrane and the aqueous medium varies significantly depending on the lipid composition and biophysical properties (Table 2). Changes in average area per lipid affect lipid packing density, which is determined by the competition between lipid headgroup repulsion and hydrophobic attraction [10]. Because they have headgroups and tails with similar cross-sectional area, major membrane lipids containing PC and PS have a cylindrical shape. Due to its large headgroup, SM has an inverted-cone shape; thus, SM lipids preferentially adopt the non-lamellar arrangement [10]. Due to their small headgroups, PE, cardiolipin, and cholesterol have a cone shape and prefer inverted nonlamellar arrangements. Membrane lipids typically self-assemble and form thermodynamically stable aggregates. As a result, any changes in this balance are expected to have an impact on the optimal area per lipid (i.e., their packing) and membrane shape [10]. Changes in lipid composition can affect the curvature and due to differences in molecular shapes and volumes of lipids, they can ultimately affect permeability of the membrane and alter the distribution of drug in membrane/aqueous systems [10]. Therefore, it is acceptable that CPT presents higher distributions in membrane models containing non lamellar assemblies of lipids (e.g., DOPE, cardiolipin) that can confer more permeable non-lamellar regions [56] than less complex models of lamellar packing like DOPC or DMPC.

**Figure 4:**
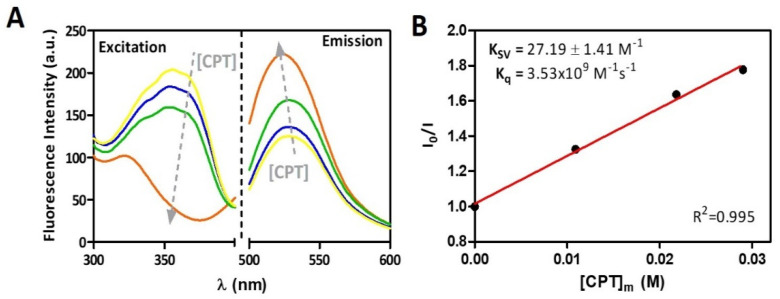
(**A**) Fluorescence excitation spectra of CPT ([CPT]_T_ of 0 to 4 × 10^−5^ M) added to cancer membrane models (3.0 × 10^−2^ M) labeled with NBD-PE probe and correspondent emission spectra of the probe (λ exc = 360 nm). (**B**) Stern-volmer plot of fluorescence quenching as a function of membrane concentrations of CPT calculated according to Equation (6).

Following the quantification of CPT distribution between the lipid and aqueous phases of the membrane model system, it is important to determine where the drug is most likely to be located within the lipid phase of the membrane. Since drug distribution was not significantly different in normal or cancer mimetic membrane models, we chose cancer mimetic membrane models to investigate drug location and assess if the drug is more superficially embedded in the phospholipid headgroups or more profoundly buried in the hydrophobic microenvironment of the phospholipid acyl chains. The CPT location in membrane was evaluated by measuring the steady-state fluorescence of the probe NBD-PE incorporated into cancer cell membrane models in the absence and presence of increasing concentrations of CPT. NBD-PE was used as a molecular ruler as previous reports have shown that contrary to other NBD-based probes, the fluorescent probe group does not project into the external aqueous phase [57] and it is well-known that NBD-PE locates at the membrane interface approximately between 19 and 20 Å from the center of the bilayer [58]. CPT is a fluorescent compound that emits between 400 and 550 nm (λ_max_ of 431 nm) when excited at 360 nm, whereas NBD-PE is a fluorescent probe that emits between 500 and 600 nm (λ_max_ of 530 nm) when excited at 465 nm. Fluorescence resonance energy transfer (FRET) was used to infer CPT location because it occurs when donor molecules (CPT) emit at shorter wavelengths that overlap with the acceptor’s absorption spectrum (NBD-PE) [57]. FRET is only sensitive for very short distances (1.5 to 6 nm) between each donor/acceptor pair [57]. As a result of the occurrence of FRET, it was possible to conclude that the donor CPT was located close to the acceptor NBD chromophores, which ultimately suggests that CPT was inserted at the membrane and close to the membrane interface, similar to NBD-PE. Figure 4A demonstrates the presence of FRET between CPT and NBD-PE.

The excitation wavelength was set to 360 nm, which is the maximum excitation wavelength for CPT. The fluorescence excitation of the donor (CPT) is decreasing in the spectra presented, and the emission intensity of the acceptor (NBD-PE) is increasing, indicating that an energy transfer is occurring from CPT to NBD-PE (Figure 4A). This means that when CPT is excited, the fluorescence emitted by CPT can excite the NBD-PE probe, which acts as the acceptor emitting fluorescence. The transfer efficiency can be determined by steady-state measurements of the extent of donor quenching due to the acceptor [25]. The extent of donor fluorescence quenching can in turn be calculated using a Stern–Volmer plot (Figure 4B) according to Equation (3). After determining the logD values of CPT in the membrane model, the effective concentrations of the drug in the membrane model system—[CPT]_m_—were calculated using Equation (4). The Stern–Volmer linear plot (Figure 4B) can be used to calculate the Stern–Volmer constant (K_SV_ = 27.19 ± 1.41 M^−1^) as well as the bimolecular quenching constant (Kq = 3.53 × 10^9^ M^−1^s^−1^) which reflects quenching efficiency or fluorophore accessibility to the quencher (Equation (5)). The obtained Kq value, is close to the reported diffusion-controlled quenching in lipid membranes, which typically results in values of Kq near 1.1 × 10^9^ M^−1^s^−1^ [25]. This also confirms that CPT is inserted at the membrane and close to the membrane interface.

### 3.3. The CPT Effect on Membrane Biophysical Properties

Aside from investigating how CPT distributes inside a membrane model, it is also critical to investigate how this drug may influence the biophysical integrity of such models. Changes in parameters like membrane fluidity and phospholipid order and/or packing play a key role in the conservation of membrane dynamics, which serves the most essential cellular functions, and changes in these parameters caused by drugs may provide useful details on their therapeutic abilities and potential toxic effects at the membrane level [9,24]. Previous logD studies have focused on model systems comprising relevant lipid mixtures to mimic drug distribution in different cell membranes (Table 2). However, we chose a pure DMPC model for studying membrane biophysical properties since it adopts the fluid lamellar phase across a broad range of hydrations and temperatures and allows simulating both organized and disordered phases found in membrane domains. Model membranes like this, which are made up of a specific class of purified lipids, are often used in vitro to mimic the behavior of biological membranes [59] [60]. These simple membrane models are a robust and repeatable platform with physical properties close to those of most cells, allowing for clear and quantitative study of phenomena in a membrane setting [59]. While these models are simplified in comparison to the mimetic models used in distribution experiments, they are a viable alternative for in vitro characterization of CPT effects in both more ordered (gel phase, Lβ) and disordered (fluid phase, Lα) membrane phases.

**Figure 5:**
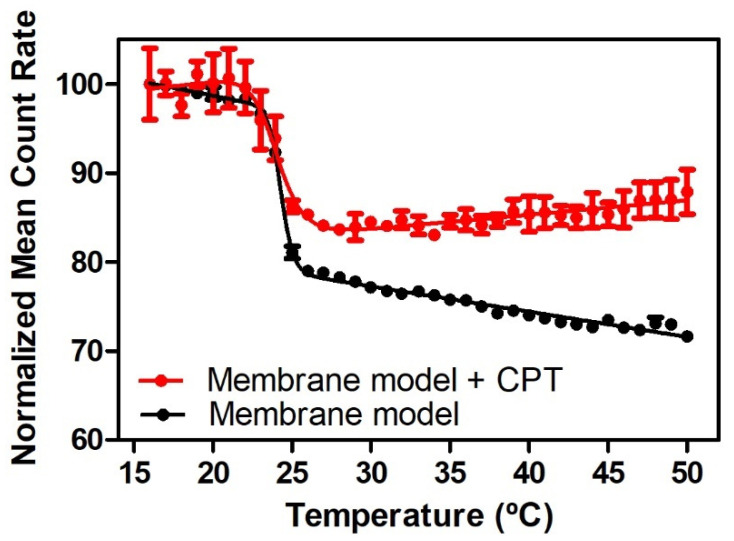
Normalized MCR of DMPC membrane models in absence (●) and in the presence of CPT (●) as a function of temperature. Each point corresponds to the mean value ± standard deviation of three experiments. Continuous lines are the best fits according to Equation (8).

There are distinct ordered phases that can disperse light of varying intensities during the lipid phase transition [61,62] As a result, within a temperature range and utilizing DLS, the average number of photons dispersed (mean count rate, MCR) by the lipid membrane system can be monitored [61,62]. This method of calculation yields a sigmoidal profile, as seen in Figure 5, which presents the results of MCR obtained with the DMPC membrane model in the presence and absence of CPT fitted by Equation (8). This equation can be used to calculate the parameters B and Tm, which describe the transition of lipid bilayers from the L_β_ to the L_α_ phase.

Tm of DMPC membrane models was determined to be 24.29 ± 0.05 °C, in agreement with the transition temperature reported for this phospholipid membrane [60]. The transition between the gel and the fluid phase of DMPC presented a B of 670.4 ± 62.21, indicating a cooperative transition, as expected for the pure lipid system. The addition of CPT to the membrane mimetic system has little effect on Tm (24.06 ± 0.16 °C), meaning that it does not provoke a membrane fluidization but does induce a significant decrease of B to 303.9 ± 42.78 (*p* < 0.001). Since the cooperative unit that undergoes the transition is primarily dominated by certain carbons, these findings point to a distribution of CPT at the polar headgroup area and at the C1–C8 level of the acyl chains of membrane phospholipids [62]. The interaction at this level explains why the main transition’s cooperativity is reduced and shows that the drug is not homogeneously dispersed inside the membrane, otherwise the phase transition will occur with high cooperativity. Nonetheless, the limited influence of CPT on membrane fluidization observed is most likely attributable to the drug’s planar nature (Figure 1), which enables it to intercalate between the phospholipids of the bilayer without disrupting its integrity significantly.

**Figure 6:**
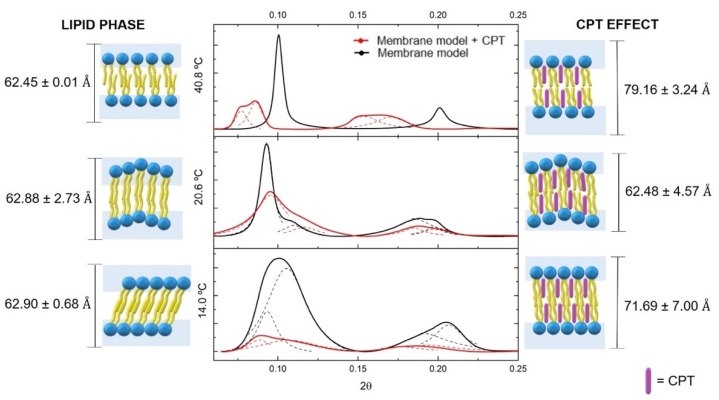
Small angle X-ray diffraction (SAXS) patterns of DMPC (black) or DMPC containing CPT (red) measured in the Lβ′ (14.0 °C), Pβ (20.6 °C), and Lα (40.8 °C) phases of DMPC. Solid lines give the best fit of the Lorentzian’ s analysis model (dashed lines) to the scattered intensities. A model of the drug–membrane interaction is proposed for each diffractogram, together with the resultant d_L_ values.

As previously stated, drug–biomembrane interactions may affect membrane biophysical properties such as fluidity and phospholipid order and/or packing. After determining that CPT has little effect on membrane fluidity, SAXS and WAXS were used to investigate the long-range bilayer order (d_L_ from SAXS) and the short-range bilayer order that determines hydrocarbon chain packing (d_S_ from WAXS) after CPT was added to DMPC bilayers. In Figure 6, are presented the SAXS diffraction patterns of DMPC in the presence and in the absence of CPT at each characteristic lipid phase: DMPC is in the gel phase (L_β’_ phase) at 14 °C; the ripple gel phase (P_β_ phase) can be found at 20.6 °C; and the DMPC bilayer is in the fluid phase (L_α_ phase) at 44.8 °C. Through viewing the graphical illustrations in conjunction with the diffraction patterns, it is possible to envision the effect of CPT in the long-range bilayer order, i.e., its effect on the thickness of the bilayer plus the water layer.

The fully hydrated DMPC bilayer alone is characterized at the L_β’_ phase by presenting a tilted bilayer with a ratio distance between Bragg peaks characteristic of lamellar packing (1:2:3). The Bragg peaks spacing was used to calculate a d_L_ of 62.90 ± 0.68 Å and a correlation length of 547.00 ± 89.35 Å.

When the drug was inserted into the bilayer, there was a significant decrease in correlation length (258.00 ± 42.02 Å, *p* < 0.05) and an increase in d_L_ (71.69 ± 7.00 Å). The membrane insertion of CPT into the bilayer at the C1–C8 level was determined using location and transition temperature studies. Thus, the increase in bilayer thickness could be attributed to the proposed CPT location, causing a change in the area requirement of the headgroups, allowing the chains to lose their tilt and be oriented upright. This stretched position of the acyl chains may account for some of the increase in d_L_ values. Indeed, when the hydrocarbon chains of the fatty acids that comprise the phospholipids are rigidly packed, C–C bonds are in an all-trans conformation, allowing the hydrocarbon chains to be accommodated in a minimum volume. The hydrocarbon chains are organized in CH_2_ groups with distances of 1.26 Å, while the distance between the C–C bonds in the terminal CH_3_ group is 1.46 Å, giving a chain length of 19.1 Å, considering the 14 C atoms of myristic acid of DMPC. This length corresponds to the hydrocarbon stretched chain, however, at the L_β’_ phase, the fully hydrated DMPC has hydrocarbon chains that are tilted by approximately 32.3° relative to the bilayer plane [63]. Given that the chains are tilted, the thickness of the bilayer will be 19.1 Å × cos (32.3°) = 16.1 Å, and the difference in thickness between the stretched and tilted hydrocarbon chains is given by 19.1 Å − 16.1 Å = 3.0 Å/monolayer, i.e., 6.0 Å/bilayer. This means that the loss of tilt caused by CPT insertion within the lipid membrane would result in a d_L_ increase of 6.0 Å. However, the addition of CPT resulted in an 8.8 Å d_L_ increase, indicating that, in addition to changing the lipid packing from pseudohexagonal (tilted) to hexagonal packing by loss of tilt angle, CPT may also induce an increase in the water layer, which will be confirmed later by WAXS measurements.

At the P_β_ phase, phospholipids in the DMPC system in the absence of CPT also lost their tilt, however, with the undulated ripple effect, two d_L_ values are observed with an average value of 62.88 ± 2.73 Å and a correlation length of 855 ± 134.68 Å. The rippled undulated phase is maintained after CPT insertion, with an average d_L_ value of 62.48 ± 4.57, which is similar to that found in the lipid membrane without the drug. The lack of changes in the d_L_ values of this phase can be explained by the fact that the phospholipids in the ripple lipid phase are less tightly packed than in the gel phase, giving a planar drug like CPT a better chance of penetrating the bilayer without disrupting the hydrocarbon chains’ characteristic packing. Nonetheless, despite maintaining the d_L_ and lipid packing, the presence of CPT in the lipid bilayer can be identified by a significant decrease in the correlation length to 431.00 ± 36.72 Å (*p* < 0.05), indicating a noticeable breakdown of the multilamellar correlation and indicating a disruption effect of this drug on the membrane structure.

The phospholipids are less ordered in the L_α_ phase, and the relaxation of hydrocarbon chains promotes phospholipid separation. As a result, the incorporation of CPT is facilitated, and the d_L_ (from 62.45 ± 0.01 Å to 79.16 ± 3.24 Å) and the correlation length (from 1549.00 ± 275.08 Å to 664.00 ± 263.68 Å, *p* < 0.01) change significantly. At the fluid phase, the increase in the d_L_ values cannot be justified by the packing change causing elongation of the hydrocarbon chain; thus, drug insertion is expected to cause an increase in the hydration layer rather than an increase in the bilayer thickness. Furthermore, the drug causes a significant decrease in correlation length during this phase as well. The overall effect of the drug decreasing correlation length observed in all lipid phases indicates a disrupting effect in the global molecular organization of the multilayer stack of the lipid bilayer.

**Figure 7:**
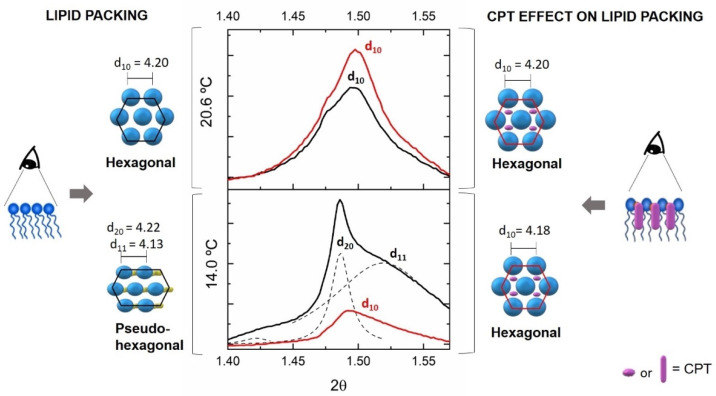
Wide angle X-ray diffraction (WAXS) patterns of DMPC (black) or DMPC containing CPT (red) measured in the L_β′_ (14.0 °C) and P_β_ (20.6 °C) phases of DMPC. Solid lines give the best fit of the Lorentzian’s analysis model (dashed lines) to the scattered intensities. A model of drug–membrane interaction is proposed for each diffractogram together with the resultant d_s_ values.

The WAXS pattern is shown in Figure 7 only for the L_β’_ and P_β_ phases, because the freedom degree of the headgroups is so high at the L_α_ phase that WAXS produces overly broad diffractograms and there are no defined d_s_ for this phase. The WAXS pattern of DMPC without drug in the L_β’_ exhibits the characteristic double Bragg peak correspond to two d_s_ values (d_20_ = 4.22 Å and d_11_ = 4.13 Å) caused by tilted phospholipids packed in a pseudohexagonal chain lattice. The addition of CPT results in a single Bragg peak, which confirms the loss of tilt effect observed by SAXS studies, resulting in a change in the lipid packing to a hexagonal chain lattice, as well as a non-significant decrease of d_s_ (d_10_ = 4.18 Å).

As observed in SAXS, the incorporation of CPT in the DMPC P_β_ phase is facilitated and did not change the lattice parameters of the DMPC headgroups, yielding a d_s_ value of 4.20 Å in both the presence and absence of CPT.

Overall, these findings suggest that CPT influences the biophysical properties of lipid membranes, particularly in the more ordered domains mimicked by the L_β’_ phase. Drug incorporation within the membrane phospholipids of these ordered phases causes loss of tilt angle, changes in lipid packing from pseudohexagonal to hexagonal lattice, and increased hydration of the headgroup region. Given that cell membrane ordered domains and lipid packing are critical for the functioning of several integrated proteins and receptors, these biophysical effects of CPT may imply membrane impairment and cell toxicity [9]. This fact justifies the need to encapsulate CPT in nanocarrier systems to avoid membrane impairment caused by free drug distribution. Indeed, our previous research showed that incorporating CPT into a nanocarrier system like SLN could preserve the membrane’s chain packing parameters [64].

### 3.4. Plasma Protein Binding of CPT and Prediction of Biodistribution Parameters

CPT biodistribution is highly dependent not only on its interaction with cell membranes, but also on its freely and bound forms present in systemic circulation. In this regard, serum proteins such as HSA function as vehicles for transporting endogenous compounds, thereby limiting the unbound fraction available for subsequent tissue distribution. As a result, studying drug affinity to HSA is also important for understanding its biodistribution and overall PK behavior.

The fluorescence of HSA is mainly due to the presence of two intrinsic fluorophores, tryptophan and tyrosine residues, and changes in HSA fluorescence are associated with its interaction or binding to a variety of quenchers. Monitoring HSA fluorescence in the absence and in the presence of a drug is a common method for determining the drug’s affinity for this plasmatic protein.

**Figure 8:**
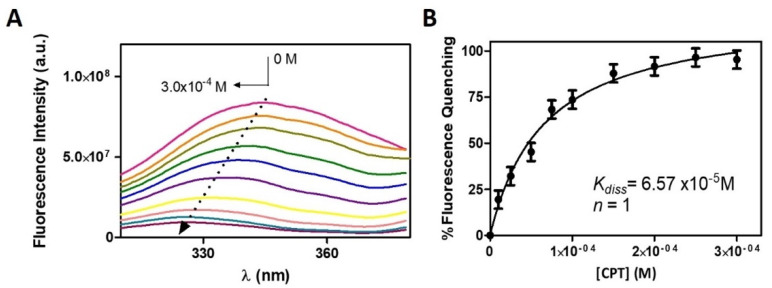
(**A**) Fluorescence spectra of HSA (2.0 × 10^−6^ M) in the presence of increasing CPT concentrations (0 to 3.0 × 10^−4^ M) at 37 °C. (**B**) Binding isotherm plot of CPT-HSA. The non-linear fit to the experimental data was made using Equation (6) and allowed to determine the dissociation constant (K_diss_) and the number of binding sites (n).

The fluorescence emission spectra of HSA solutions in the presence of increased amounts of CPT are shown in Figure 8.

As the CPT concentration increased, there was a clear decrease in the intensity of the HSA fluorescence emission (fluorescence quenching). A shift in the maximum emission band to shorter wavelengths is also visible as a result of the fluorescence quenching. Both observations point to a strong interaction between CPT and HSA, with the possibility of a complex formation. Emission spectral shifts can be interpreted as a change in the surrounding hydrophobicity of the chromophore because fluorescence emission is highly dependent on the local microenvironment. Typically, hypsochromic shifts (or blue shifts) are associated with decreased polarity, indicating that HSA complexation with CPT has resulted in the formation of a hydrophobic environment around the tryptophan and tyrosine residues.

Similar observations were noticed by Yang et al., when they studied the binding of CPT to BSA. In the same study, the authors collected information provided by several methodologies about the drug–membrane binding and concluded that the formation of the CPT-BSA complex occurs mainly through electrostatic interactions and hydrogen bonds. To the fluorescence quenching data, a non-linear fitting was applied (Equation (6)) from which it was possible to determine that CPT binds to a single HSA site (n = 1) with a binding constant (K_bind_ = 1.52 ± 0.26 × 10^4^ M^−1^) and a negative value of Gibbs free energy (ΔG_bind_ = −5.93 ± 0.10 kcal∙mol^−1^), suggesting that drug and serum protein associate spontaneously (Equation (7)). The HSA–CPT binding constant falls within the range of previously published binding constants of CPT in carboxylate or lactone form for the interaction of HSA with CPT (5.5 × 10^3^ to 1.2 × 10^6^ M^−1^) [13] and is also close to the binding constant reported by Li et al., for the interaction of CPT with BSA (K_bind_ = 3.72 × 10^4^ M^−1^) [17]. Based on K_bind_ values, a PPB of 91.95% (Equation (14)) was calculated, indicating that the majority of CPT is bound to HSA, leaving only 8.05% of the free drug available for distribution to the tissues. The magnitude of these values indicates that CPT and HSA have a strong interaction [25].

Additional biodistribution parameters of CPT were also determined using the values of PPB to HSA and logD values (Table 2 normal cells) obtained. The unbound CPT fraction in plasma and tissues were respectively determined as f_u_ = 0.081 (Equation (13)) and f_ut_ = 0.0054 (Equation (15)). These parameters were used to obtain VD_SS_ = 5.75 ± 0.93 L·Kg^−1^ (Equation (12)).

## 4. Discussion

CPT is expected to communicate with a variety of biological interfaces immediately after oral administration, raising obstacles to its biodistribution [9,46]. Relying on in silico calculations like the Rule of 5 stated by Lipinski for orally administered drugs, it would be possible to conclude that CPT presents favorable oral absorption [35]. Moreover, as the PSA value of CPT is less than 140 Å^2^ (PSA_CPT_ = 79.73 Å^2^), the molecule accomplishes one of the prerequisites to be absorbed in gastrointestinal tract (GIT) [36]. Still, following the BCS scheme of drug classification, CPT shows characteristically a poor aqueous solubility and permeability that commonly leads to a poor oral absorption. Additionally, along the GIT, the drug will experience a pH gradient that will cause its hydrolysis originating an anionic carboxylate form. It is well described in the literature that non-ionized drugs are more readily absorbed along the GIT [27,65], thus the anionic form of the drug will reduce its membrane permeability, consequently reducing its absorption. The contradictory results in the absorption potential of CPT highlight the fallibility of using solely in silico molecular calculations as tools to predict ADMET profile of drugs. ADMET modeling used for PK drug prediction relies heavily on the logP parameter estimated from theoretical distribution of drugs in octanol/water systems, along with charge/ionization. However, in the case of polar ionizable drugs, like CPT, a simple theoretical computational calculated logP in octanol/system would not represent the interactions that the drug can establish with polar membrane headgroups. We therefore propose that reliable modeling should not only be based on in silico computational predictions but should instead be combined with in vitro logD experimental determination studies in the membrane/water systems, as this model more closely represents the polar and non-polar membrane environment and better translates the binding established with polar drugs. Accordingly, CPT presented a logD value of 2.89 ± 0.23 concerning its distribution in membrane models mimetic of normal cells which is typical of moderate lipophilic molecules (0 < logD < 3). The moderate lipophilic CPT profile can also lead to non-specific binding to mucin hydrophobic domains, potentially reducing its diffusion in GIT mucus [66]. Thus, its encapsulation into drug delivery systems (DDS) can be a good approach to improve CPT aqueous solubility, maintaining the stability of the molecule across the GIT, and protecting the drug from mucin interaction and improving the drug delivery after crossing the mucous layer. logD values are also important in deciding the more adequate type of DDS and strong relations have been found between drug lipophilicity and DDS hydrolipidic balance [67]. Based on the determined logD, CPT is more compatible with micelles and albumin nanoparticles [67].

After considering GIT absorption, it is important to consider other physiological barriers like plasma proteins. As the drug enters the systemic circulation, it may be exposed to plasma proteins, where their primary purpose is to transport exogenous molecules across the body. If the drug–plasma protein interaction is not balanced and reversible, biodistribution problems may be triggered due to the either too low or too high affinity of a drug to plasma proteins like HSA [68]. From the fluorescence quenching assay, a binding constant of 1.52 ± 0.26 × 10^4^ M^−1^ was determined which is indicative of a strong interaction between CPT and HSA [25]. Additionally, a PPB value was calculated as 91.95%, meaning that a major part of CPT in solution is bounded to HSA, leaving only 8.05% of free CPT available to be distributed into the tissues. When free CPT leaves systemic circulation, mutual associations with various biological interfaces continue to occur until CPT reaches the therapeutic target. The logD obtained in the membrane model of normal cells, suggests capacity to permeate cell membranes and low metabolic tendency [27,69]. The lipophilicity properties of CPT can also influence its biodistribution and bioaccumulation in different tissues. Regarding this, the logD value was also used to estimate the CPT bioaccumulation. It was shown that CPT is more likely to bioaccumulate in adrenal glands (61.44%), thyroid (30.72%), kidneys (3.07%), and heart (3.07%). In addition to the predicted bioaccumulation of CPT in non-target tissues, the ASI, which explains the drug’s distribution on the adipose tissue, was also determined (2.53 ± 0.12), suggesting a high tendency to be bioaccumulated in the fat tissue [28]. Moreover, the unbound CPT fraction in plasma and tissues, f_u_ = 0.081 and f_ut_ = 0.0054, were respectively determined. From these parameters, it was possible to calculate the *VD_SS_* value (5.75 ± 0.93 L·Kg^−1^), which can be translated in a high volume of distribution [70]. The high VDss value obtained agrees with the high ASI value obtained. Indeed, drugs with VD_SS_ values between 1 and 5 L·Kg^−1^ are characterized as having large volumes of distribution due to affinity for adipose tissues which hampers their elimination from the body. Moreover, the VD_SS_ value calculated from in vitro logD and PPB values is in agreement with the in vivo VD_SS_ value of 5.16 ± 1.25 L·Kg^−1^, obtained from the reported value of 190 ± 46 L·m^−2^ [71], considering the average values of body surface (1.9 m^2^) and body weight (70 kg).

Considering the high volume of distribution, as well as the bioaccumulated sites, including the ASI value, it is possible to conclude that CPT will face potential clearance problems. Because of the compound’s tendency to accumulate in fat tissue and the high value of VDss, the CPT half-life can be enhanced, reducing its elimination rate, and its excretion from the body is hindered [70,72]. Biodistribution problems can also result from an undesirable distribution of CPT into the brain. BBB is a high selective membrane that separates the systemic circulation from the central nervous system. Drugs that pass through this membrane can lead to toxicity issues. CPT distribution in a model mimicking the BBB lipid composition suggested a high affinity of this drug to the BBB endothelial membrane (LogBB = 2.76 ± 0.06). This high affinity can constitute a major problem and once again, some strategies should be considered to control the biodistribution of CPT, such as its encapsulation into DDS that can more specifically direct the drug to the target tissues.

CPT as an anticancer agent has to be able to selectively permeate through cancer cells. The logD in the cancer cell membrane model was determined as 3.08 ± 0.22, suggesting a high distribution of CPT molecules in such membranes. Membrane location studies were performed to determine, whereas CPT locates at the membrane level of cancer cells using the NBD-PE probe that is located at 20 Å from the phospholipid bilayer core sensing the hydrocarbon and membrane interfacial location. FRET indicated the drug’s proximity to the probe, and the Kq value indicate that CPT distributes into the lipid phase near the interfacial region. DLS studies, which pointed to a CPT location within the C1–C8 region of the membrane, and SWAXS, which indicated the effect of CPT increasing the hydration layer at the membrane interface and penetration within the headgroup phospholipid region, changing lipid packing even in the more ordered phases, corroborate this information.

Gathering all the obtained information, it is possible to identify potential issues in the oral administration of CPT at absorption level and, even when it is absorbed, its large biodistribution can result in potential lack of selectivity. This lack of selectivity for the therapeutic target is also demonstrated by the lack of significant differences in logD of CPT in the cancer cell and normal cell membrane models. Therefore, and as previously referred, encapsulation of CPT in DDS could present a promising alternative to overcome the here reported pharmacokinetic issues.

Various DDS have been developed to improve solubility and lactone stability of CPTs, including micelles, liposomes, and nanoparticles [73,74]. Micelles are macromolecular assemblies formed by the aggregation of amphiphilic molecules that have charged or charge-neutral hydrophilic head groups at the water interface and hydrophobic chains toward the vesicle’s center, forming a spherical structure. The structure’s hydrophobic interior allows for efficient encapsulation of hydrophobic molecules, such as the CPT, for drug delivery. Micelles formed from block copolymers containing PEG chains have proven useful because they avoid binding to albumin [75,76] which is a significant barrier to CPT distribution, as demonstrated by the high PPB predicted in our biodistribution profiling. Kawano et al., for example, produced micelles from block copolymers of PEG and poly(aspartic acid) esterified with the benzyl group [76]. The micelles released nearly half of the CPT loaded after 24 h, but blood plasma levels were 150× higher than free CPT. Furthermore, when compared to the free drug, tumor levels in mice models showed an 8-fold increase in CPT when using the micelles. Almeida et al. have also synthesized an amphiphilic block copolymer from PEGylated chitosan and oleic acid [77]. The polymeric micelles formed have CPT encapsulation efficiency of 78% and are able to stabilize the lactone form in up to 75% of CPT. Furthermore, the PEG chains will be used to create a stealth nanosystem capable of evading HSA binding. It has also been demonstrated that CPT released from the chitosan-based micelles is pH dependent, which may favor oral administration [77]. As a result, these chitosan-based micelles with lipid moieties for CPT encapsulation and polymeric coatings may be of interest not only for stealth purposes (to reduce PPB and increase distribution), but also to promote a balance of GIT mucoadhesion and mucopenetration upon a putative oral administration.

Liposomal drug delivery has received a lot of attention for delivering a variety of insoluble therapeutics, including CPT. Burke et al. identified the need for an alternative route to deliver CPT and discovered that when drugs are noncovalently complexed with liposomes, lactone stability increases [78]. In vivo made of bis(dodecyl)benzoic acid and PEG with an HSA coating yielded a CPT encapsulation efficiency of 80% [79]. After a 2.5 mg∙Kg^−1^ dose of CPT, blood plasma levels increased dramatically from an AUC value of 1.1 μg∙h∙mL^−1^ for the CPT solution to an AUC value of 24.8 μg∙h∙mL^−1^ for the HSA-coated liposome. Biodistribution studies in mice with C26 colon carcinomas using CPT loaded liposomes revealed a nearly 10-fold increase in tumor accumulation as well as a 60-fold increase in blood plasma at 8 h when compared to the tumor accumulation seen when using the free drug. Because our biodistribution profiling predicted an accumulation of CPT in several off-target tissues, encapsulating the drug in liposomes or other nanosystems could provide a more specific delivery method. Indeed, nanosystems can exhibit specific EPR accumulation in cancer tissues, or they can enable surface functionalization with ligands specific to target tissues, or they can be formed by components that are responsive to physiological stimuli, triggering drug release only in tissues where these stimuli are present. He et al., for example, developed a redox-triggered CPT liposomal system for improved tumor therapy [80]. Because of the significantly higher glutathione level, tumor cells have a reductive intracellular milieu. As a result, when CPT liposomes are internalized into tumor cells, glutathione cleavage of the disulfide bonds in the liposomes promotes the release of active CPT for maximum therapeutic effect [80].

Lipid nanoparticles are another interesting alternative for the delivery of CPT. Martins et al. investigated the ability of solid lipid nanoparticles (SLN) to deliver CPT into the brain parenchyma after crossing the blood–brain barrier [64]. In vivo biodistribution studies of intravenous CPT-loaded SLN in rats revealed that CPT loaded had significantly higher brain accumulation when compared to the non-encapsulated drug. PK studies also revealed that when CPT was encapsulated in SLN, its deposition in peripheral organs was reduced, resulting in a reduction in potential side toxicological effects [64]. The increased circulation of CPT enclosed in SLN has proven to be a huge benefit, which could be attributed to the coating of SLN with non-ionic surfactants that provide steric hindrance. This strategy can reduce PPB while increasing CPT circulation in the body. Furthermore, increased circulation increases brain LDL receptor exposure to the SLN with the incorporated drug inside, which will most likely result in increased CPT accumulation in the brain parenchyma. Alternatively, the increased amount of circulating SLN loaded with CPT may not cross the BBB but may simply serve to deliver high concentrations of CPT to the luminal surface of BBB cells, establishing a local high concentration gradient between blood and brain that increases passive drug diffusion through the BBB [81]. This is especially important in the case of CPT, as we predicted that it has BBB permeation potential.

Despite the fact that this set of in silico and in vitro models is a simple and useful approach for predicting PK issues of drug candidates, it is important to stress that the model systems used have some limitations in that they do not capture the entire complexity of biological membranes or the rich microenvironment of tissues. Indeed, drug–phospholipid interactions are also influenced by proteins and nutrients [82]. Drugs can have an impact on cell function by altering the activity of transport proteins. Drugs can also interfere with intracellular metabolic processes by inhibiting or activating enzymes [9]. Protein regulation via drug–membrane interactions can eventually result in changes in cell signaling and gene expression, which may interfere with the pathological state [9]. In any case, simplification of the in vivo condition is unavoidable and necessary for analyzing specific molecular interactions at the membrane level that could be useful for understanding drug biological behavior, as well as, for optimizing the properties of drugs and nanocarriers capable of coping with PK challenges of anticancer therapeutics such as CPT.

## Figures and Tables

**Table 1:** In silico prediction of several physicochemical descriptors using the CPT chemical structure on Chemaxon^®^ software.

MW (g∙mol^−1^)	PSA (Å^2^)	VWSA (Å^2^)	logP	S (mg∙mL^−1^)	pKa	H Donors	H Acceptors
348.36	79.73	441.88	1.52	0.0559	3.07 11.71	1	4

Abbreviations: MW—molecular weight; PSA—polar surface area; VWSA—Van der Waals surface area; logP—log_10_ of the octanol/water partition coefficient; S—intrinsic aqueous solubility; pKa—negative log_10_ of the ionization constant.

**Table 2:** Distribution coefficients of CPT obtained in a biphasic membrane/aqueous system.

Membrane Model	pH	Composition	Method	LogD
Cancer cells membranes	5.8	DOPC (25%), CHOL (15%), EPC (31.8%), DOPS (17%), DOPE (8%), Cardiolipin (2.5%), SM (0.7%)	Derivative UV–Vis spectroscopy	3.14 ± 0.13 ^ns^
			Fluorescence spectroscopy	3.01 ± 0.31 ^ns^
Normal cells membranes	7.4	DOPC (45%), DOPE (20%), DOPS (20%), CHOL (10%), SM (10%)	Derivative UV–Vis spectroscopy	2.78 ± 0.28 ^ns^
			Fluorescence spectroscopy	2.63 ± 0.15 ^ns^
		DMPC or DMPG [16,45,46]	Fluorescence anisotropy	2.00 ± 0.16 ***
		DOPC [47]		1.55 ± 0.05 ***
		DOPG [47]		1.97 ± 0.05 ***
		Octanol: water [18,47,48]	Fluorescence spectroscopy	1.73 ± 0.08 ***
BBB endothelial membrane	7.4	PC (12.6%), PE (33.1%), PI (4.1%), PS (18.5%) and PA (0.8%)	Derivative spectroscopy	3.64 ± 0.15 ***

ns: Comparisons between derivative UV-Vis spectroscopy and fluorescence spectroscopy or between cancer cell and normal cells model used in this work were performed using two-way ANOVA with the Sidak’s multiple comparisons test and indicate no statistical significance. ***: Comparisons between BBB and normal cells model used in this work or between normal cells model used in this work and normal cells model reported in the literature were performed using one-way ANOVA and indicate statistical significance (*p* < 0.05).

## Data Availability

The data presented in this study are available on reasonable request from the corresponding author.
